# Alterations of the marginal soft tissue (gingival margin) following periodontal therapy: A clinical study

**DOI:** 10.4103/0972-124X.55843

**Published:** 2009

**Authors:** Ira Gupta, K. L. Vandana

**Affiliations:** *Senior Lecturer, Department of Periodontics, Rama Dental College and Hospital, Kanpur, India*; 1 *Senior Professor, Department of Periodontics, College of Dental Sciences, Davangere, India*

**Keywords:** Crown lengthening, curettage, flap surgery, gingival margin position, scaling and root planing

## Abstract

**Background and Objectives:**

The evaluation of gingival margin position (GMP) plays a vital role in periodontal therapy and is critical in esthetic/plastic surgical procedures revolving around restorative dentistry. Comparative evaluations of GMP measurements in various periodontal therapies are scarce. Thus, the objectives of this study are to measure the alteration in the gingival margin position following various therapies, and to compare GMP alterations among different treatment modalities from the baseline to six months after therapy.

**Materials and Methods:**

The changes in GMP were studied for MB, B, DB, ML, and L sites for SRP, curettage, and flap surgery, and for MB, B, and DB sites for crown lengthening cases at the end of one, three, and six months after therapy. The results were interpreted from baseline to one, three, and six months posttreatment.

**Statistical Analysis:**

The results were subjected to statistical analysis. Paired ‘*t*’-test was used for intra-group comparisons and intergroup comparisons were done by one-way ANOVA.

**Results:**

The GMP changed from baseline in all the sites at different time periods following various therapies. The net results after six months were an apical shift of GMP in SRP, curettage, and flap surgery, and a coronal shift of GMP in crown lengthening.

**Conclusion:**

GMP shows various patterns of alteration after various periodontal therapies. One should wait for the GMP to become stable before attempting any restorative procedure.

## INTRODUCTION

In recent years, several reports have been presented describing the short- and long-term effects of various forms of periodontal therapy.[1] Previous studies have indicated that different surgical procedures may promote varying degrees of tissue reorganization during healing.[2] It was also found that recession took place following a treatment program that involved both surgical and nonsurgical measures, which led to various aesthetic and sensitivity problems.[3,4] Also, the final location of the gingival margin position (GMP) has a major role to play in cases of crown lengthening procedures.[5] However, there is no way to know at what point of time the gingival margin will achieve a stable position. A waiting period of six months prior to definitive crown placement has been suggested in one study, whereas another study proposes tooth preparation immediately after epithelialization of the attached gingiva.[6] The evaluation of GMP plays a vital role in periodontal therapy and is critical in esthetic/plastic surgical procedures revolving around restorative dentistry.[7] A medline search was done with the key words, “Periodontal flap”, “Gingival margin position”, short-term (six months), long-term (>1 year), and it revealed a limited number of articles, many of them pre 1980 and surprisingly, no articles for crown lengthening procedures. Thus, the aim of the present study is to evaluate dimensional changes such as GMP after periodontal flap and crown lengthening surgeries. The objectives of this study are to measure and compare the alteration in the gingival margin position sitewise, from baseline to six months following SRP, curettage, flap surgery, and crown lengthening.

## MATERIALS AND METHODS

For this study, subjects belonging to both the sexes were selected from the outpatient Department of Periodontics. The age group of the selected patients ranged from 16 to 45 years. The inclusion criteria were patients with minimal 20 teeth who required scaling and root planing (SRP), curettage, periodontal flap surgery, and crown lengthening, and who were willing to follow the recall checkup. Exclusion criteria consisted of patients who had undergone periodontal therapy in the past six months and who had gingival recession; those with history of known systemic diseases, pregnant and lactating women, and smokers. All examinations were performed by one trained examiner[8–11] who was not involved in any aspect of the therapy. The clinical parameters recorded for the selected subjects included Modified gingival index (Lobene, Weatherford, Ross 1986) and GMP at baseline, one, three, and six months.

A customized stent was fabricated for each subject to record the GMP. All the measurements were made by the same investigator to the nearest 0.5 mm[12] throughout the study period. The features of the flap surgery consisted of the flap being raised with crevicular incision; no osseous surgery was done. The flap was positioned back without any displacement. Gingivectomy was the method of choice for the crown lengthening procedure. The changes in GMP were studied in mesio-buccal (MB), mid-buccal (B), disto-buccal (DB), and mid-lingual (L) sites for scaling and root planing, curettage, flap surgery, and in mesio-buccal, mid-buccal, and disto-buccal sites for crown lengthening cases. These measurements of GMP were repeated at one, three, and six months postoperatively. After completion of different periodontal therapies, the patients were enrolled in a supervised maintenance care program including “professional tooth cleaning” once every two weeks during a six months' period.[2]

### Statistics

The measurement of data thus collected at different time periods were subjected to statistical analysis. Paired ‘*t*’-test was used to make intragroup comparisons of measurements and the changes in GMP from baseline to 1-3 and six months posttherapy.

## RESULTS

This study assessed the alteration in the GMP (in mm) following scaling and root planing, curettage, flap surgery, and crown lengthening. In the present study, a total of 636 sites were available for study from 13 patients, which included 169 SRP sites, 105 curettage sites, 290 flap surgery sites, and 72 crown lengthening sites. In the SRP group, there was a net apical shift of GMP by the end of the six months, with means and standard deviations of −0.59±0.60, −0.50±0.65, −0.29±0.92, −0.19±0.70, −0.25±0.72 for MB, B, DB, ML, and L sites respectively. This apical shift was highly significant (*P* < 0.001) for MB and B sites, significant for lingual sites (*P* < 0.05) and not significant for DB (*P* = 0.09) and ML (*P* = 0.11) sites [Table 1]. In the curettage group, the decrease in apical shift of GMP continued further in all the sites six months after therapy, with means and standard deviations of −0.33±0.46, −0.33±0.51, −0.31±0.60, −0.40±0.66, −0.29±0.51 for MB, B, DB, ML, and L sites respectively. The differences were statistically significant (*P* < 0.01 for MB and B, *P* < 0.05 for DB, ML, L) for all the sites [Table 2].

**Table 1:** Sitewise comparison of GMP changes in SRP over time

Sites	Baseline	1 month	*P* value /*t* value	Baseline	3 months	*P* value / *t* value	Baseline	6 months	*P* value /*t* value
MB	4.63	5.06	<0.001 H.S	4.63	5.30	<0.001 H.S	4.63	5.22	<0.001 H.S
	1.30	1.32	(−)6.59	1.30	1.38	(−)7.13	1.30	1.15	(−)5.58
B	5.77	6.15	<0.001 H.S	5.77	6.26	<0.001 H.S	5.77	6.27	<0.001 H.S
	1.90	1.87	(−)8.67	1.90	1.85	(−)7.65	1.90	1.86	(−)4.42
DB	4.61	4.85	0.1 N.S	4.61	4.90	0.05 S	4.61	4.90	0.09 N.S
	1.69	1.64	(−)1.72	1.69	1.57	(−)2.01	1.69	1.40	(−)1.76
ML	4.38	4.65	<0.001 H.S	4.38	4.77	<0.001 H.S	4.38	4.57	0.11 N.S
	1.07	1.10	(−)4.10	1.07	1.02	(−)3.88	1.07	0.97	(−)1.64
L	5.31	5.54	<0.05 S	5.31	5.67	<0.01 S	5.31	5.56	<0.05 S
	1.39	1.47	(−)2.69	1.39	1.30	(−)3.04	1.39	1.37	(−)2.08

* Paired ‘t’-test *P* < 0.05: Significant, (S) H.S.: Highly significant, N.S.: Nonsignificant, (−): Indicates apical shift GMP given in mm

**Table 2:** Sitewise comparison of GMP changes in curettage over time

Sites	Baseline	1 month	*P* value /*t* value	Baseline	3 months	*P* value / *t* value	Baseline	6 months	*P* value / *t* value
MB	4.90	5.31	<0.001 H.S	4.90	5.29	<0.01 S	4.90	5.24	<0.01 S
	1.61	1.56	(−)5.46	1.61	1.50	(−)3.51	1.61	1.53	(−)3.35
B	5.79	6.29	<0.001 H.S	5.79	6.19	<0.01 S	5.79	6.12	<0.01 S
	1.57	1.55	(−)4.58	1.57	1.49	(−)4.00	1.57	1.47	(−)3.01
DB	4.31	4.74	<0.01 S	4.31	4.69	<0.01 S	4.31	4.62	<0.05 S
	1.47	1.24	(−)4.08	1.47	1.12	(−)3.34	1.47	1.13	(−)2.36
ML	4.52	5.10	<0.001 H.S	4.52	4.88	<0.05 S	4.52	4.93	<0.05 S
	1.50	1.37	(−)6.61	1.50	1.09	(−)2.31	1.50	1.18	(−)2.79
L	5.26	5.76	<0.001 H.S	5.26	5.64	<0.01 S	5.26	5.55	<0.05 S
	1.63	1.51	(−)8.37	1.63	1.31	(−)3.07	1.63	1.40	(−)2.55

* Paired ‘t’-test *P* < 0.05: Significant (S) H.S.: Highly significant (−): indicates apical shift GMP given in mm

In the flap surgery group, the GMP showed a reduction in apical shift by the end of six months relative to the one and three months' measurements, with means and standard deviations of −0.29±0.68, −0.40±0.69, −0.31±0.72 for MB, B, and DB sites respectively. The difference was highly significant in all the sites (*P* < 0.001) except for DB sites where the difference was significant (*P* < 0.01) [Table 3]. In the crown lengthening group, the GMP showed increased coronal shift for MB sites by the end of six months, with a mean and standard deviation of 0.73±0.72, and reduction in coronal shift for B and DB sites with means and standard deviations of 0.25±0.63 and 0.85±0.87 respectively. This difference was relative to three months and was highly significant for MB and DB sites (*P* < 0.001) but not significant for B sites (*P* = 0.06) [Table 4]. The alteration in the GMP at the end of six months was appreciated as either an apical shift of GMP from the baseline (negative values), coronal shift of GMP from the baseline (positive values), or no change at all. The groups showed some apical as well as coronal shift of GMP from the respective baselines [Table 5]. The comparison between the alterations in GMP in periodontal therapies showing apical shift at various time periods, indicated that SRP, curettage, and flap surgery showed a net apical shift of GMP relative to the baseline. The variation in the pattern of alteration of GMP between the groups by the end of six months was not significant (F = 0.29 and *P* = 0.75) [Table 6].

**Table 3:** Sitewise comparison of GMP changes in flap surgery over time

Sites	Baseline	1 month	*P* value /*t* value	Baseline	3 months	*P* value /*t* value	Baseline	6 months	*P* value /*t* value
MB	5.03	5.72	<0.001 H.S	5.03	5.76	<0.001 H.S	4.75	5.04	<0.01 S
	1.71	1.95	(−)6.46	1.71	1.95	(−)7.04	1.44	1.52	(−)3.33
B	5.57	6.28	<0.001 H.S	5.57	6.31	<0.001 H.S	5.36	5.76	<0.001 H.S
	1.81	2.11	(−)6.73	1.81	2.13	(−)6.94	1.66	1.81	(−)4.51
DB	5.17	5.91	<0.001 H.S	5.17	5.86	<0.001 H.S	4.63	4.94	<0.01 S
	1.96	2.18	(−)7.83	1.96	2.05	(−)8.03	1.34	1.43	(−)2.97
ML	4.80	5.43	<0.001 H.S	4.80	5.54	<0.001 H.S	4.53	4.93	<0.001 H.S
	1.48	1.60	(−)7.48	1.48	1.40	(−)8.73	1.34	1.25	(−)3.70
L	5.15	5.72	<0.001 H.S	5.15	5.86	<0.001 H.S	4.84	5.38	<0.001 H.S
	1.62	1.71	(−)8.26	1.62	1.56	(−)9.69	1.37	1.36	(−)6.15

* Paired ‘t’-test *P* < 0.05: Significant (S) H.S.: Highly significant (−): Indicates apical shift GMP given in mm

**Table 4:** Sitewise comparison of GMP changes in crown lengthening over time

Sites	Baseline	1 month	*P* value /*t* value	Baseline	3 months	*P* value /*t* value	Baseline	6 months	*P* value /*t* value
MB	4.97	4.67	<0.01 S	4.84	4.33	<0.01 S	4.88	4.15	<0.001 H.S
	1.22	1.55	(+)2.79	1.28	1.62	(+)3.91	1.39	1.67	(+)4.95
B	6.20	6.09	0.21 N.S	6.22	5.95	<0.05 S	6.15	5.90	0.06 N.S
	1.11	1.32	(+)1.28	1.09	1.40	(+)2.65	1.14	1.48	(+)1.96
DB	4.79	4.43	<0.01 S	4.55	3.83	<0.001 H.S.	4.54	3.69	<0.001 H.S
	1.38	1.54	(+)3.65	1.39	1.26	(+)4.96	1.49	1.22	(+)4.83

* Paired ‘t’-test *P* < 0.05: Significant (S) H.S.: Highly significant N.S.: Nonsignificant (+): Indicates coronal shift GMP given in mm

**Table 5:** Distribution of total number and percentage of sites with GMP changes six months after different therapies

Diff. (mm)	SRP			Curettage			Flap surgery			Crown lengthening		
				
	Sites with different measurement			Sites with different measurement			Sites with different measurement			Sites with different measurement		
				
	*n*	Percentage (%)		*n*	Percentage (%)		*n*	Percentage (%)		*n*	Percentage (%)	
−3	0	0.0	59.8	0	0	55.2	1	0.3	56.1	0	0.0	9.7
−2.5	1	0.6		0	0		1	0.3		0	0.0	
−2	2	1.2		0	0		7	2.4		0	0.0	
−1.5	10	5.9		4	3.8		27	9.3		0	0.0	
−1	34	20.1		20	19.0		43	14.8		2	2.8	
−0.5	54	32.0		34	32.4		84	29.0		5	6.9	
0	44	26.0		33	31.4		82	28.3		15	20.8	
0.5	11	6.5	14.2	12	11.4	13.3	29	10.0	15.5	22	30.6	69.5
1	9	5.3		2	1.9		13	4.5		17	23.6	
1.5	3	1.8		0	0		3	1.0		8	11.1	
2	0	0.0		0	0		0	0.0		1	1.4	
2.5	0	0.0		0	0		0	0.0		1	1.4	
3	1	0.6		0	0		0	0.0		0	0.0	
4	0	0.0		0	0		0	0.0		1	1.4	
Total	169	100		105	100		290	100		72	100	

**Table 6:** Comparison of alteration in GMP in three different periodontal therapies at various time intervals

		SRP	Curettage	Flap surgery	ANOVA*
0-1	Mean	−0.31	−0.48	−0.66	F = 17.6
Month	SD	0.50	0.40	0.75	*P* < 0.001, HS
0-3	Mean	−0.44	−0.38	−0.72	F = 15.4
Month	SD	0.63	0.55	0.74	*P* < 0.001, HS
0-6	Mean	−0.36	−0.33	−0.39	F = 0.29
Month	SD	0.73	0.54	0.72	*P* = 0.75, NS

* One-way ANOVA

## DISCUSSION

It has been reported that the treatment of periodontal disease often results in recession of the gingival margin and in the exposure of coronal portions of the root surfaces, leading to various aesthetic and sensitivity problems, and the coronal growth of the gingival margin.[13,14] In the present study, the term, “gingival margin position” refers to either apical, coronal, or no change in the gingival margin following various periodontal therapies. The use of the term, “Gingival Margin Position” (GMP) is very important as very few authors report the changes in the position of the gingival margin as an apical shift of the gingival margin[15] or marginal soft tissue[5] or the soft tissue margin[16] The changes in the GMP were recorded from baseline to six months after therapy as studies have shown that the major alterations resulting from active therapy occur within the first six months following therapy—the healing phase.[13,17,18] In the present study, there was no control group that received either no treatment or oral hygiene instruction alone. However, even if a control group of patients with untreated periodontal disease is lacking in this study, the data reported here are still valid in the sense that they illustrate the effect of treatment on the parameters traditionally used to describe periodontal health and disease.[19] Measurements using the stents appear to be more reliable than subgingival CEJ readings.[20,21]

The GMP is interpreted as an apical shift (−), a coronal shift (+), or as no change () depending upon the measurement difference between baseline to a specific time period. Several studies reported in periodontal literature often refer to the apical shift of GMP as recession.[2,10,13,17] However, only the apical shift exposing roots, needs to be considered, in the true sense, as gingival recession. The changes in GMP behave differently over time with different periodontal therapies, *i.e*., apical shift/coronal shift seen in the early months (< 3 months) can remain the same or change after 1-3 months, and may get stabilized by six months.[17] The results of the study are as follows: The apical shift of GMP in SRP posttreatment was in accordance with various studies.[3,4,9,10,13] Most of the apical shift of GMP took place in the first three months in SRP, in accordance with a study by Badersten *et al.,* in which most of the recession was seen in the first 2-3 months. The proximal surfaces showed the greatest amount of recession after nonsurgical therapy of moderately advanced periodontitis cases.[22] In a similar study by Badersten *et al.* of treatment of severely advanced periodontitis cases, it was seen that limited gingival recession occurred during the initial three months and more notably, recession took place until a level of 1.6 and 1.8 mm recession was reached at the 12 months' examination.[3] Using CEJ as the fixed reference to measure gingival recession after a single episode of root planning, Proye *et al*. reported that the gingival margin was 0.72 mm coronal to the cementoenamel junction. Significant gingival recession was found to occur at the one week time point, moving the gingival margin 0.16 mm coronal to the cementoenamel junction. There were no significant differences at subsequent time points; however, the initial recession was maintained in accordance to the baseline values.[10] The term, “gingival recession” is appropriate when the roots are exposed, however, the use of words, ‘coronal to CEJ’ is confusing in the above study. Hughes *et al.* have also reported changes in the height of gingival margin one month after scaling, by using CEJ as the fixed reference point to measure the gingival margin location. It was seen that 1 mm of gingival recession took place in almost ¼ of all cases, and there was a greater tendency for recession of the mesial gingival margin after scaling with increasing severity of the initial inflammation.[9] While comparing root planing and surgical treatment, Isidor *et al.* reported that presurgical treatment resulted in a mean recession of the gingival margin (patient mean) of 0.6-0.8 mm. At the three months' follow-up examination, 1.8 mm of gingival recession was seen in root planing-treated areas. After six months following treatment, the gingival recession was identical to that observed three months after treatment.[4] While studying the dimensional alteration of the periodontal tissues following six different treatment modalities including SRP alone, Lindhe *et al.* measured the distance from the stent to the gingival margin. They reported that the recession occurred between the baseline and the six months' examinations for sites which had an initial probing depth of 4 mm and that the healing resulted in a more pronounced gingival recession in buccal sites than at approximal sites.[13] Although the American Academy of Periodontology did not include gingival curettage as a method of treatment in its Guidelines for Periodontal Therapy, the procedure has not been abandoned in day-to-day practice. The surgical elimination of shallow edematous pockets is commonly treated either with SRP and/or curettage alone. Gingival margin position measurements are restricted to SRP and surgical cases such as MWF with and without osseous reduction; no time curettage is mentioned in literature. The present study evaluated GMP in curettage cases as curettage is one of the pocket elimination procedures.[23] In curettage, the apical shift of GMP seen six months after therapy was significant at all the sites (*P* < 0.05, *P* < 0.01). At different time intervals of this study, the apical shift at DB site was consistent as compared to MB, B, ML, and L sites where the apical shift of GMP was highly significant at one month (*P* < 0.001) and significant (*P* < 0.01, *P* < 0.05) at three and six months respectively. No studies have been reported in literature that could help to compare the results of the present study.

This study did not show any gingival recession due to exposure of the root. Most of the studies reported in the literature do not discriminate between mere apical shift, which occurs commonly, and actual gingival recession. Two distinct changes were observed in GMP in this study. In the first three months, both apical and coronal shift of GMP increased following flap and crown lengthening respectively. The apical shift of GMP reduced in flap surgery cases at the end of six months, suggesting a possible coronal shift of GMP. In crown lengthening cases, there was a consistent increase in the coronal shift of GMP. The GMP showed no change in 28.3% of flap surgery sites and 20.8% of crown lengthening sites. About 56.1 and 30.6% of sites showed a maximum of 0.5 mm apical and coronal shift in flap surgery and crown lengthening cases respectively. In flap surgery, the apical shift of GMP was appreciated in 29% of sites from baseline to six months posttherapy. The apical shift was highly significant (*P* < 0.001) in B, ML, and L sites and significant (*P* < 0.05) in MB and DB sites. The apical shifts were consistently significant in MB, B, DB, ML, and L sites at different time intervals of this study; the results are similar to previous studies.[13,4] Periodontal literature reports a few studies on the location of the gingival margin following surgical crown lengthening. Various therapies studied for crown lengthening include APF[14] or APF with osseous recontouring[18] and gingivectomy.[6] These different modalities showed variations in the patterns of alteration of GMP. Hence, to avoid any such variation in our study, the cases selected for crown lengthening were those that had been treated with gingivectomy without osseous recontouring.

In this study, the coronal shift of GMP was consistent over the study period in MB and DB sites, although the B sites showed an inconsistent pattern of alteration of GMP. No study was found in literature to compare the results of our study. Various reasons have been stated for the alteration of GMP following various periodontal therapies. Thorough scaling and root planing of the tooth promotes resolution of the gingival inflammation, permitting shrinkage and recession of the “unattached” gingiva.[9] With increasing severity of the inflammation, there was a greater tendency for gingival recession because of the increasing degree of edema associated with the enlargement and distention of the tissue commonly seen in severe gingivitis. This initial difference seen in the extent of recession between various treatment modalities, diminishes over time due to a coronal rebound of the soft tissue margin following surgical treatment.[11,24] Lindhe and Nyman (1980) found that the buccal gingival margin shifted to a more coronal position (about 1 mm) after apically repositioned flap procedure during 10-11 years of maintenance.[5] In interdental areas that were denuded following surgery, Van der Velden (1982) found an upgrowth of around 4 mm of gingival tissue three years after surgery.[14] However, no clear reasons have been mentioned in their study for the coronal upgrowth.

The clinical implication of the measurement of GMP in periodontics is that the tooth requiring restorative therapy may need to undergo any of the chosen periodontal therapies. Hence, the alteration of GMP should be understood so that restoration or other periodontal therapy such as root coverage procedures can be attempted at the right time. The term, “GMP” should be used during the follow-up period rather than simply using the term, “Gingival recession.” Future studies should include GMP changes in restorations like crowns and abutments.

## CONCLUSION

GMP changes over time and stability of the gingival margin position are critical in assessing the outcomes of periodontal therapy and are important for restorative dentistry. Before attempting any restorative procedure, one should wait for the GMP to become stable as the decision-making for periodontal and restorative procedure relies on the stability of GMP.
